# Pure Food

**Published:** 1859-05

**Authors:** 


### PURE FOOD.

It is no economy to use inferior food. It is a saving of money, and time and health, to give a higher price for what we eat, if it be fresh and perfect, than to obtain it for less on account of its being wilted, or old, or partially decayed.

Some people prefer to make their meat tender by keeping, which means that decomposition is taking place; in plainer phrase, it is rotting. Such meats require less chewing, and may appear very tender, but it is a physiological fact, that they are not digested as easily or as quickly as solid fresh meat.

When a vegetable begins to wilt, it is no longer that vegetable, because a change of particles has taken place, and in such proportion it is unnatural—it is dead—and to eat it tends to death.

One of the most horrible forms of disease is caused by eating sausages which have been kept a long time; more common in Germany than elsewhere. Scarcely anything saddens us so much in passing through some of the bye streets and the more eastern avenues, as the sight of the long kept meats and shrivelled vegetables, which are sold to the unfortunate poor at the corner Dutch groceries.

But the poverty stricken are not the only sufferers, the richest men come in for their share, for themselves and for their families in proportion, as the mistresses of their splendid mansions are incompetent or inattentive to those household duties, the proper performance or neglect of which makes all the difference between a true wife and a contemptible doll.

With all the high sounding advantages of high sounding "Young Ladies' Boarding Schools," and "Institutes" and all that, with all the twaddle about learning French and German, and music and æsthetics, how many of these paint-like girls are any more fit to take charge of a man's household than to navigate a ship, or calculate a parallax. Does one in a million of them know the philosophy and uses ot that now indispensible article of household furniture, a refrigerator. If taken to Bartlett's, on Broadway, how many of them can tell why he places the ice on the top of his polar refrigerator, and by so placing gives the greatest cold to the articles below; why it is there is no wood on the inside to become saturated with dampness and the fumes of butter and lard and milk and meats; why it is that a particular kind of metal of a particular shape, and by the aid of ten pounds of ice a day, will give a large family all the ice water needed, and will keep the bottom and sides and area of the refrigerator dry, by attracting all the dampness to a particular spot, and by an interior arrangement gives no dark corners for dirt, but makes the whole as light as day; and thus combining dryness, coolness and cleanliness every article is kept fresh and perfect for any reasonable time. The study of an article of a practical nature of this kind will give a "young lady" about to be married a better idea of the philosophy of things of this sort than she has learned from all the books skimmed over or learned by rote, or mere memory, through her whole "course" of study, and would save her husband more money than without that knowledge she is worth; for the woman who does not know how, and does not make it her business, to take care of what her husband brings into the house, is not worth a button, even if she could smatter a dozen languages, dance every indecent polka ever devised, and play all the tunes in the music book.

The study of milk, its nature, qualities and uses, might well be made a branch of education in every school for girls. Studied aright it is a fruitful and very extensive field of most interesting investigation; i. e. The nature of swill milk and that of the pure article. The difference between that of the common cart and of the Rockland association. How long milk remains pure. What part of any vessel of milk is richest, and what part the most inferior, and why. Why it is that warming milk or freezing it, decomposes it and changes its nature. How to keep it in its pure state for a long time together. The knowledge of these things on the part of our wives would save the money and promote the happiness and health of every family in the land.

